# Structure-Based Design of Acetolactate Synthase From *Bacillus licheniformis* Improved Protein Stability Under Acidic Conditions

**DOI:** 10.3389/fmicb.2020.582909

**Published:** 2020-10-27

**Authors:** Ting Zhao, Yuan Li, Siqi Yuan, Yang Ye, Zhifu Peng, Rongqing Zhou, Jun Liu

**Affiliations:** ^1^Faculty of Bioengineering, Wuliangye Liquor College, Sichuan University of Science and Engineering, Yibin, China; ^2^Wuliangye Group Co. Ltd., Yibin, China; ^3^College of Biomass Science and Engineering, Sichuan University, Chengdu, China

**Keywords:** acetolactate synthase, site-directed mutagenesis, tetramethylpyrazine, acetoin, and Chinese liquor, acidic stability

## Abstract

Catabolic acetolactate synthase (cALS) plays a crucial role in the quality of liquor because of its ability to catalyze the synthesis of the endogenous precursor product α-acetolactate of the aromatic compound tetramethylpyrazine (TTMP) and acetoin. However, the vulnerability of cALS to acidic conditions limits its application in the Chinese liquor brewing industry. Here we report the biochemical characterization of cALS from *B. licheniformis* T2 (BlALS) that was screened from Chinese liquor brewing microorganisms. BlALS showed optimal activity levels at pH 7.0, and the values of *K*_m_ and V_max_ were 27.26 mM and 6.9 mM⋅min^–1^, respectively. Through site-directed mutagenesis, we improved the stability of BlALS under acidic conditions. Replacing the two basic residues of BlALS with acidic mutations (N210D and H399D) significantly improved the acid tolerance of the enzyme with a prolonged half-life of 2.2 h (compared with wild-type BlALS of 0.8 h) at pH 4.0. Based on the analysis of homologous modeling, the positive charge area of the electrostatic potential on the protein surface and the number of hydrogen bonds near the active site increased, which helped BlALS^N210D–H399D^ to withstand the acidic environment; this could extend its application in the food fermentation industry.

## Introduction

**FIGURE 1:**
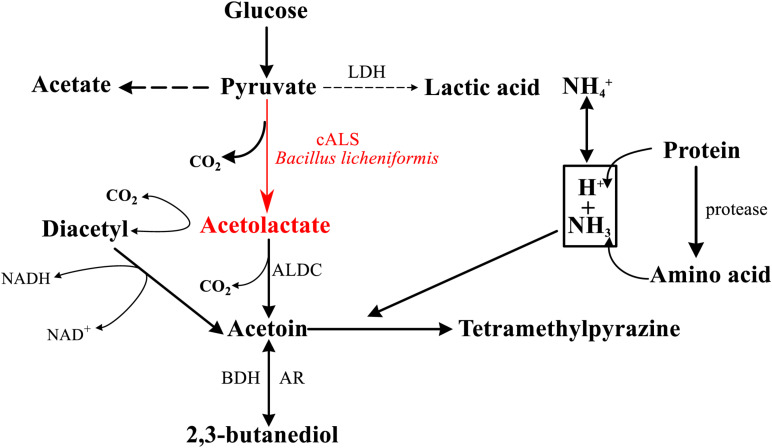
General scheme of pathway for the formation of TTMP in *Bacillus sp.* cALS and its product are highlighted in red (cALS, acetolactate synthase; LDH, lactate dehydrogenase; ALDC, α-acetolactate decarboxylase; BDH, 2,3-butanediol dehydrogenase; AR, acetoin reductase).

Tetramethylpyrazine (TTMP), which is a compound commonly found in sauce-flavored liquor and sesame-flavor liquor, is not only an important compound related to the flavor of Chinese liquor, but also a health-care factor, that endows Chinese liquor with healthy functions (Kao et al., 2006). The main pathway for the production of TTMP in fermentation processes of Chinese liquor has been confirmed, which was a breakthrough for the improvement of liquor quality (Zhu et al., 2010). *Bacillus licheniformis* produces TTMP with glucose as a raw material by its own multi-step enzymatic reaction (Figure 1; Fan et al., 2007; Zhu et al., 2010), and the biosynthesis is in need of a key enzyme, acetolactate synthase (ALS, EC 2.2.1.6) (Jia et al., 2017).

Acetolactate synthase belongs to the family of thiamine diphosphate (ThDP)-dependent enzymes, which is involved in α-acetolactate biosynthesis. Relying on the catalysis of ALS, pyruvate is decarboxylated to form a cofactor-bound hydroxyethyl group, which is transferred to a second molecule of pyruvate to obtain α-acetolactate; it is a key intermediate in various metabolic pathways of microorganisms, which has a wide range of uses in industrial production (Pang et al., 2004). ALS consists of Flavin adenine dinucleotide (FAD)-dependent anabolic acetohydroxyacid synthase (AHAS) and FAD independent catabolic acetolactate synthase (cALS), but cALS is only found in some bacteria (Renna et al., 1993; Lee et al., 2015).

Catabolic acetolactate synthase has an efficient ability to synthesize acetoin, and it can be used as a potential regulatory node for genetic engineering to produce high-value downstream products (Xiao and Lu, 2014; Fan et al., 2018). Therefore, controlling the expression, stability, and catalytic activity of cALS in microorganisms during liquor fermentation in the brewing process could increase the yield of acetoin and 2,3-butanediol and, thereby, improve the flavor and healthy attributes of liquor. As reported, several recombinant strains have been developed by optimizing the expression levels of cALS, such as *B. licheniformis* (Huo et al., 2018) and *Bacillus subtilis* (Renna et al., 1993). cALS from different bacteria showed different biochemical characterizations. cALS from *B. subtilis* and *Lactococcus lactis* were neutral enzymes, and their optimal pH values were 7.0 and 6.5–7.0, respectively, except that their *K*_m_ values were different, 13.6 and 70 mM, respectively, (Snoep et al., 1992; Atsumi et al., 2009). The optimal pH of cALS from *Klebsiella pneumoniae* and *Leuconostoc mesenteroides* were 5.8 and 5.3, respectively, and optimal K_m_ values were 8.0 and 10 mM, respectively, (Peng et al., 1992; Phalip et al., 1995). Therefore, we hope to screen strains of high-expressing cALS from the *Daqu* of Wuliangye’s strong flavor liquor to improve the enzymatic activity of cALS and to control effectively the flavor of the expression levels of liquor.

Daqu is a raw material used for making Chinese Baijiu. It is made by crushing barley, wheat, peas, etc., and adding water to make it look like a brick; various microorganisms in nature are then allowed to grow on it. It is a source of microbial fermentation in liquor and is crucial in the fermentation process. *Bacillus* is one of the main species of aroma-producing substances in the medium-temperature Daqu (MTD) (Xiao et al., 2017). Due to the acidic environment in the fermentation process of liquor, the activity of WT cALS from *Bacillus* is unstable, which also limits its application in liquor fermentation (Zhu and Xu, 2010). Therefore, it is desirable to enhance the acid tolerance of cALS from *Bacillus*, which originated from MTD by genetic mutations to improve the yield of TTMP.

In this work, we focused on cALS from *B. licheniformis* T2 (BlALS) that was obtained from MTD in our laboratory. The alsS gene sequence for expressing cALS was cloned, and the BlALS was purified to determine and to improve its biochemical characterization. To adapt to an acid environment, we improved the acid tolerance of BlALS by site-directed mutagenesis based on a structural model to achieve the accumulation of the precursor for generating more health factors and aroma compounds in the production of acetoin, 2,3-butanediol, and TTMP. According to biochemical and structural analysis, the acidic mutations N210D and H399D were helpful for BlALS^N210D–H399D^ to withstand an acidic environment. The results of the enzyme activity test showed that the acid tolerance of BlALS^N210D–H399D^ was improved significantly. This study further proved the feasibility of directed mutation of an industrial enzyme based on protein structure and provides reference for industrial applications.

## Materials and Methods

### Strains, Plasmids, and Chemicals

The strains and plasmids used in this study are shown in Supplementary Table S1 as Supplementary Data. *B. licheniformis* T2 was screened from MTD by our laboratory. *Escherichia coli* strains DH5α and BL21 (DE3) were used for the plasmid amplification and protein purification, respectively. The plasmid pEGX-6p-1 was used for the induced expression. The cloning vector pMD19-T, T4 DNA ligase, and restriction enzymes were purchased from TaKaRa (Japan). ThDP and FAD were purchased from Shanghai Yuanye Biotechnology (Shanghai, China). The Fast Mutagenesis System was purchased from TransGen Biotech (Beijing, China).

### Cloning Genes and Site-Directed Mutagenesis

The sequence of *B. licheniformis* T2 *alsS* was cloned into the pEGX-6p-1 vector for expression in *E. coli* DE3. The *alsS* open reading frame was amplified from *B. licheniformis* T2 by PCR, using primers with *Bam*HI and *Xho*I sites. The recombinant strain was selected by resistance to ampicillin. Site-directed mutagenesis on the *alsS* was carried out by using the overlapping PCR-based method (Horton et al., 1990; Supplementary Table S2). The successful introductions of the desired mutations were confirmed by sequencing.

### Expression and Purification of BlALS

*Escherichia coli* BL21 (DE3) cells that carried the pEGX-6p-1-*alsS* expression vector were grown in LB medium that contained 50 mg/mL ampicillin at 37°C, and BlALS overexpression was induced by adding IPTG to a final concentration of 0.2 mM when OD_600_ reached 0.7–0.8. The cells were further grown for 8 h at 25°C and harvested by centrifugation. The bacterial pellet was re-suspended by adding PBS buffer (140 mmol/mL NaCl, 2.7 mmol/mL KCl, 10 mmol/mL Na_2_HPO_4_, 1.8 mmol/mL KH_2_PO_4_, pH 7.0). The harvested cells were lysed by sonication and centrifuged to remove the cell debris. The supernatant was collected and incubated with Glutathione Sepharose 4B slurry at 4°C for 4 h, and then the incubated mixture was transferred to a Glutathione Sepharose 4B column. Impurities and target proteins were eluted separately using three times the column volume of 1X PBS and two times the column volume of elution buffer (10 mM reduced glutathione dissolved, 50 mM Tris–HCl, pH 8.0), respectively. The GST-tag of the target protein was removed by digestion in the glutathione Sepharose 4B column with PP enzyme overnight at 4°C. The molecular weight of the BlALS was confirmed by SDS-PAGE analysis (12% separation gel), which was stained by Coomassie Brilliant Blue. The protein concentration was detected by the Bradford method using BSA as a standard protein. Finally, the target protein was stored in PBS buffer with pH of 7.0 at −80°C.

### Measurement of BlALS Activity

Enzymatic activity was determined based on the decarboxylation of acetolactate at 37°C to form an acetoin molecule that reacted with creatine and an alpha-naphthol solution under alkaline conditions to form a red substance. The enzymatic activity of BlALS was determined according to a previously reported method (Singh et al., 1988). Briefly, 1 mL reaction buffer (100 mmol/L potassium phosphate buffer that contained 40 mmol/L sodium pyruvate, 1 mmol/L magnesium chloride, 1 mmol/L ThDP, and 10 μmol/L FAD, pH 7.0) and 20 μL BlALS were added into centrifuge tubes, followed by incubation at 37°C for 10 min. The enzymatic reaction was terminated by adding 50 μL of 3 mol/L H_2_SO_4_ and incubated at 37°C for 25 min for the decarboxylation of acetolactate. Next, 250 μL 1.7% (w/v) α-naphthol solution and 250 μL of 0.17% creatine (w/v) were added, and then the solution was incubated at 37°C for 30 min. The resulting sample was measured at 525 nm using a UV spectrophotometer. One unit of BlALS activity was defined as producing 1 μmol acetolactate in 1 min. The standard curve equation for acetoin is *y* = 0.0602*x* + 0.0955, *R*^2^ = 0.9951, where *x* is the concentration of acetoin (μmol/L) and y is the OD_525_ value. Kinetic parameters of BlALS were determined by measuring enzymatic activity under different pyruvate concentrations. The Michaelis constant (*K*_m_) and the maximum reaction rate (V_max_) values of the substrate were obtained according to a Non-linear fitting.

The optimum pH of BlALS activity was determined using pyruvate as a substrate in 100 mM sodium acetate buffer (pH 4.0-6.0), 100 mM phosphate buffered saline buffer (PBS buffer, pH 7.0-8.0), and 100 mM glycine NaOH buffer (pH 9.0-11.0). The effect of pH on BlALS stability was measured by placing the purified enzyme in different elution buffers on ice for 10 h. The optimal temperature was detected by performing the standard assay at temperatures that ranged from 20 to 60°C in 100 mM sodium phosphate buffer (pH 7.0). Residual activity was tested by standard assays.

### Fermentation of the Recombinant *E. coli* DE3

The recombinant *E. coli* strain was cultured in LB medium with 50 μg/mL ampicillin at 37°C for 16 h in a shake flask. The shake flask culture (250 mL) was then transferred into a 3 L bioreactor that contained 2.5 L fermentation medium, which consisted of 40 g/L glucose, 10 g/L peptone, 1 g/L yeast powder, 1 g/L K_2_HPO_4_⋅3H_2_O, 0.7 g/L MgSO_4_⋅7H_2_O, 0.0003 g/L FeSO_4_⋅6H_2_O, 0.005 g/L MnSO_4_⋅6H_2_O, and 5 g/L NaCl. Initial pH was adjusted to 6.8-7.0, and 72 h batch fermentation was conducted. Ventilation was 0.4 vvm, rotation speed: 0–6 h, 200 rpm; 6–24 h, 300 rpm; and 24–72 h, 400 rpm. The quantification of cell biomass and glucose consumption was performed by corresponding ultraviolet spectrophotometry, and the concentrations of acetoin and diacetyl were measured every 12 h over the fermentation period. BlALS overexpression was induced by adding IPTG to a final concentration of 0.2 mM when OD_600_ reached 0.8–1.0. Cell concentration was monitored at 600 nm, and the dry cell weight (DCW) was determined by a pre-calibrated relationship (Xu et al., 2013). The supernatant was used to determine total sugar using the 3,5-dinitrosalicylate method as described by Miller (Miller, 1959). Determination of acetoin and diacetyl concentration was by GC-MS.

### Construction of 3D Model

The protein sequences of BlALS were submitted to the SWISS-MODEL Workspace that built relatively accurate structural models (Biasini et al., 2014). The crystal structure of cALS from *B. subtilis* (PDB ID: 4RJJ) was selected as the template model, which shared a 75.86% sequence identity with BlALS in 97% coverage. Figures were prepared using PyMOL (Delano, 2002).

## Results

### Cloning, Expression, and Purification of BlALS

**FIGURE 2:**
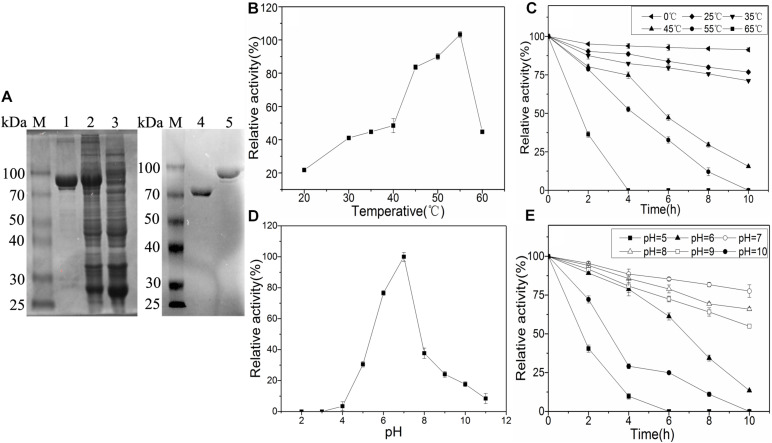
Characterization and enzymatic properties of WT BlALS. Average values from three independent experiments are presented, and all of the measurements of the enzyme activities were measured in three parallels. **(A)** SDS-PAGE analysis of purified BlALS in *E. coli*. M: Protein Marker; Lane 1: Purified BlALS; Lane 2: The total intracellular proteins of *E. coli*. DE3/pGEX-6p-1-*alsS* induced by IPTG; Lane 3: The total intracellular proteins of *E. coli*. DE3/pGEX-6p-1 induced by IPTG; Lane 4: Purified BlALS without GST tag; Lane 5: Purified BlALS. **(B)** Optimum temperature was examined by detecting BlALS activity at various temperatures that ranged from 20 to 65°C. **(C)** Thermal stability of the purified enzymes was determined by pre-incubation at different temperatures from 0 to 65°C, and then by detecting the residue activity under standard assay conditions. **(D)** Optimum pH was decided by detecting WT BlALS activity at 37°C (setting pH range: 2.0–11.0). **(E)** Stability analysis of WT BlALS by incubating BlALS at different pH values (5.0–10.0) at 0°C.

*Bacillus licheniformis* was isolated from MTD by our laboratory. After sequencing, the *alsS* gene had a 1719 bp open reading frame that encoded a protein of 572 residues (accession NO. WP_095324682.1). Sequence alignment between the *alsS* gene expression product and BlALS showed 100% protein sequence similarity. As expected, the molecular weight of the target protein obtained was about 90 kDa (BlALS was about 64 kDa, and GST was about 26 kDa) on SDS-PAGE, and the molecular weight of the protein that removed the GST tag was the same as expected (Figure 2A). The purified BlALS and BlALS^N210D–H399D^ were diluted to 0.313 mg/mL for measurement of enzymatic activity, and their specific activities were 22.71 +2.01 and 22.27 +1.93 U/mg, respectively. As expected, the specific activity of BlALS^N210D–H399D^ was similar to that of WT BlALS, and there was no significant difference between the specific activity measured in this study and that reported by Huo et al. (2018).

### Enzymatic Properties of Recombinant BlALS

To obtain the optimum temperature and pH of BlALS, enzymatic activity was measured under different temperatures (0–65°C) and pH (2.0–11.0). The optimal temperature for BlALS activity was 45–60°C with a maximum activity at 55°C at pH 7.0 (Figure 2B). BlALS activity retained nearly 90% at 0°C, and about 70% at 25–35°C after 10 h, and rapid inactivation was observed at >45°C (Figure 2C). The optimal pH for the activity of BlALS was 7.0 (Figure 2D). When the pH range was outside of 4.0–7.0, activity of BlALS decreased rapidly until it was inactivated completely at pH 2.0–3.0. After 10 h of pre-incubation, >50% of the enzymatic activity was retained at pH 7.0–9.0, but only 22.5% of the enzymatic activity was retained at pH 6.0 for 10 h, and then it decreased more rapidly at pH 5.0 after 2 h (Figure 2E). However, some cALS exhibited highly efficient activity under an acidic environment, such as the cALS expressed from *K. pneumoniae* and *L. mesenteroides*, where optimal activity was obtained at pH 5.8 and 5.3, respectively (Peng et al., 1992; Phalip et al., 1995). The existence of acidophilic homologs provided the possibility that acid tolerance of BlALS was modified.

The kinetics of BlALS was detected using pyruvate as a substrate at different concentrations under optimal pH and temperature. *K*_m_ and V_max_ values were determined by non-linear fitting analysis. BlALS and BlALS^N210D–H399D^ catalyzed the synthesis of α-acetolactate from two molecules of pyruvate. The *K*_*m*_ and V_max_ of BlALS were 27.26 +1.1975 mM and 6.899 +0.102 mM⋅min^–1^ (Supplementary Figure S1A). In addition, the *K*_*m*_ and V_max_ of BlALS^N210D–H399D^ were 26.91 +1.4189 mM and 6.886 +0.12 mM⋅min^–1^ (Supplementary Figure S1B), respectively, and there was no significant difference between BlALS and BlALS^N210D–H399D^.

### Modeling and Site-Directed Mutagenesis of BlALS

The structure of BlALS was modeled as stated above. The model estimation precision of the GMQE was 0.88, the QMEAN was 0.36, and the MolProbity Score was 0.92, which was calculated using the SWISS-MODEL Workspace. These relatively ideal scores indicated the models had correct fold and the correct topology, which ensured the rationality of the predicted model.

**FIGURE 3:**
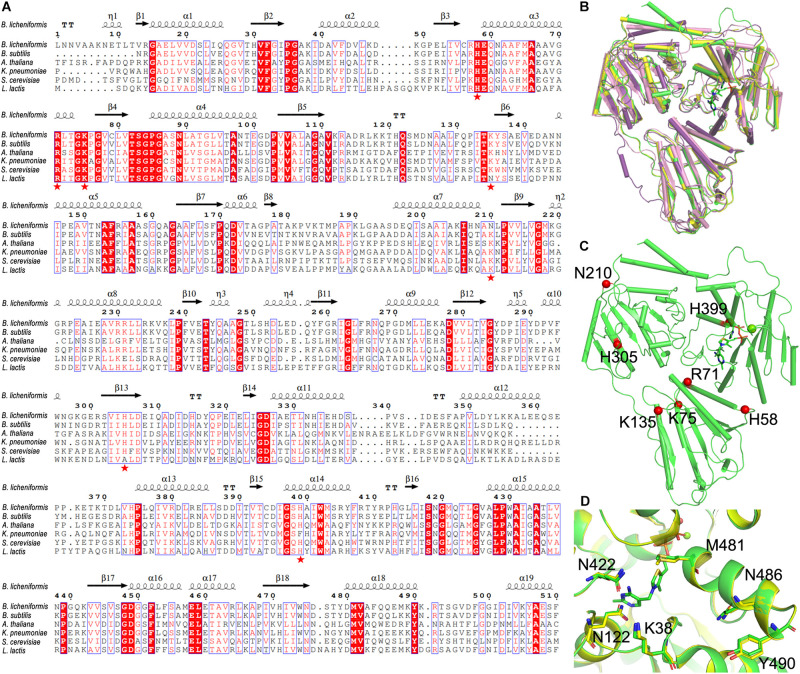
Selection of mutated residue. **(A)** Multiple sequence alignment of BlALS of *B. licheniformis* compared with other bacterial cALS homologs. Red shading and red words represented identical and similar residues, respectively. The selected mutant residues (H58, R71, K75, K135, N210, H305, and H399) were marked with red pentagons. **(B)** Structure comparison of the BlALS and homologs from other specials (*A. thaliana* (PDB ID 1Z8N), violet; *Bacillus subtilis* (PDB ID 4RJJ), yellow; *K. pneumoniae* (PDB ID 1OZG), pink) using superimposition. **(C)** Positions of the selected mutant residues are depicted in the BlALS model. BlALS is shown as a cartoon in green, and the Cα atoms of mutant residues are illustrated as red spheres. **(D)** The residues near the active center are shown as stick models.

By aligning to the known structures of cALS from other species (*B. subtilis*, PDB ID: 4RJJ; *Arabidopsis thaliana*, PDB ID: 1Z8N; *K. pneumoniae*, PDB ID: 1OZG; *Saccharomyces cerevisiae*, PDB ID: 1T9B) (Pang et al., 2004; McCourt et al., 2005; McCourt et al., 2006; Sommer et al., 2015), our results showed that BlALS was significantly conserved in the predicted active center: (i) the K38 and Q485 of BlALS participated in the catalytic mechanism, as confirmed in cALS from *K. pneumonia* (Pang et al., 2004); (ii) the Q122, Q422, M481, and Y490 of BlALS was important to the accurate localization of ThDP to promote the generation of the second condensation reaction of acetyl-lactic acid, as confirmed in cALS from *B. subtilis* (Sommer et al., 2015). These residues were conservative in sequence and conformation (Figure 3D), which further verified the reliability of the predicted model.

The conventional method of improving acid tolerance of recombinant protein is to convert its alkaline residue into an acidic residue (Roy et al., 2012). Based on the results of sequence alignment (Figure 3A), we selected the potentially valuable amino acid residue mutation sites (Figure 3C). The highly conserved sequences of H58, R71, K75, K135, and H305 were replaced by aspartic acid, and the mutants were named as BlALS^H58D^, BlALS^R71D^, BlALS^K75D^, BlALS^K135D^, and BlALS^H305D^. In addition, N210 and H399 were also relatively conservative, which may mean that they had greater variability, so they were mutated to obtain BlALS^N210D^, BlALS^N210E^, and BlALS^H399D^. According to the results of homologous structures and sequence alignment, we designed eight single point mutations (BlALS^H58D^, BlALS^R71D^, BlALS^K75D^, BlALS^K135D^ BlALS^N210D^, BlALS^N210E^, BlALS^H305D^, and BlALS^H399D^) for a subsequent acid tolerance test.

### Enzymatic Stability of Mutants at Low pH

**FIGURE 4:**
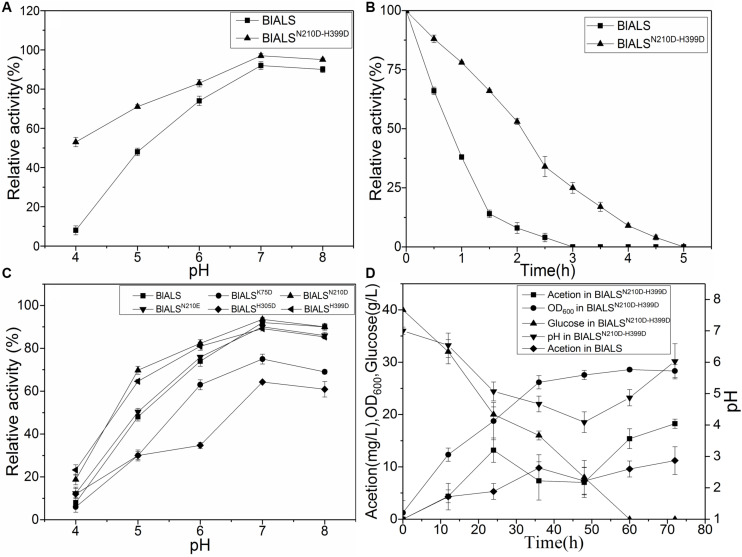
The comparison of stabilities between WT BlALS and mutants at different values of pH. Average values from three independent experiments are presented, and all of the measurements of the enzyme activities were measured in three parallels. **(A)** Enzyme stability of BlALS^N210D–H399D^ and WT BlALS at pH range from 4.0 to 8.0. **(B)** Half-life of BlALS^N210D–H399D^ and WT BlALS at pH 4.0. **(C)** Effect of pH (ranged from 4.0 to 8.0) on enzyme stability of WT BlALS and the mutants. **(D)** Fed-batch fermentation of recombinant *E.coli* for acetoin production.

To test the acid tolerance of the mutants mentioned above, the mutants were incubated for 2 h at pH 4.0–8.0, and their activity was measured (Figure 4C). For the single mutants, BlALS^H58D^, BlALS^R71D^, and BlALS^K135D^ were completely ineffective. Besides, the enzymatic activity of BlALS^K75D^ and BlALS^H305D^ also decreased significantly compared with WT BlALS. However, the enzymatic activity of BlALS^N210D^, BlALS^N210E^, and BlALS^H399D^ was similar to that of WT BlALS at pH 6.0–8.0. Remarkably, at pH 4.0 and pH 5.0, the residual activity of BlALS^N210D^ was 18.77 and 23.12%, respectively, and for BlALS^H399D^ it was 69.77 and 64.60%, respectively, which were higher than that of WT BlALS under the same conditions (Figure 4C). The results implied that acid tolerance of BlALS^N210D^ and BlALS^H399D^ had improved. To further optimize acid tolerance of BlALS, we constructed a double site mutation BlALS^N210D–H399D^, and it showed 53 and 71% activity at pH 4.0 and pH 5.0, respectively (Figure 4A). It should be emphasized that the enzymatic activity at pH 4.0 increased significantly, and the half-life of enzymatic activity was also prolonged from 0.8 to 2.2 h (Figure 4B), which indicated that acid tolerance of BlALS^N210D–H399D^ improved significantly.

### Comparing the Ability of Acetoin Production by Fermentation

To evaluate the potential of the mutant strain to produce acetoin, recombinant *E. coli* was inoculated in a 3L fermentation medium. After 72 h of fermentation, the concentration of acetoin in the bioreactor for WT and mutant strains were 11.20 and 18.24 mg/L, respectively, (Figure 4D). Compared with the WT strain, the recombinant *E. coli* that contained BlALS^N210D–H399D^ produced more acetoin. The accumulation of precursor acetoin contributed to the formation of TTMP during the fermentation stage of liquor (Zhu et al., 2010). Therefore, in the acid environment of liquor fermentation, BlALS^N210D–H399D^ with better acid tolerance increased the concentration of TTMP in liquor, which improved the aroma and quality of these fermented products. The results of the fermentation experiment showed that acetoin had a transient peak at 24–36 h, began to decrease at 24–48 h, and then the concentration of acetoin began to rise. pH also showed a similar trend, which reached its lowest value at 48 h and then rose slowly. According to Xiao’s report, acetoin can be used as an energy source to maintain the normal metabolism of bacteria (Xiao et al., 2009). We speculate that at 24–48 h, due to the large consumption of glucose and the increase in bacteria, the energy supply in the fermentation broth was insufficient, and acetoin was consumed as an energy substance. While the concentration of BlALS and BlALS^N210D–H399D^ was too low to produce enough acetoin, the consumption rate of acetoin was greater than its production rate, so the concentration of acetoin decreased gradually. After 48 h, the concentrations of BlALS and BlALS^N210D–H399D^ were enough, and the formation rate of acetoin was higher than the consumption rate, so the concentration of acetoin began to increase (Holtzclaw and Chapman, 1975). As expected, the strain containing BlALS^N210D–H399D^ had more advantages in acetoin production. Many studies have shown that with an increase in BlALS and BlALS^N210D–H399D^ activity, cells began to synthesize a large amount of acetoin (Martin et al., 2005). At this time, acetoin played the role of neutralizing intracellular acidic products to maintain intracellular pH (Xiao and Xu, 2007), which was consistent with the results of a gradual increase in pH after 48 h. Although the growth density of mutant strain was slightly lower than that of wild-type strain due to the change of metabolic pathway, there was no significant difference in expression level of BlALS and BlALS^N210D–H399D^ (Supplementary Figure S2). However, the acetoin yield of mutant strain was significantly higher than that of wild-type strain, which further indicated that the mutant had stronger ability to produce acetoin during fermentation because of the improvement of acid tolerance.

## Discussion

BlALS from the bacterium *B. licheniformis* synthesized α-acetolactate during the fermentation stage of liquor in an acidic environment (pH 4.0–6.0). WT BlALS was too poor in its acid tolerance (i.e., deactivated rapidly when the pH was <6.0) to satisfy an industrial application. Fortunately, the mutant BlALS^N210D–H399D^ had significantly improved acid tolerance compared with WT BlALS and a higher yield of acetoin. As shown in the results for enzymatic activity and fermentation, the relative enzymatic activity of WT BlALS and BlALS^N210D–H399D^ was calculated at pH 4.0 and pH 5.0, respectively, (Figure 4B), and the half-life of the enzyme at pH 4.0 extended from 2.2to 0.8 h (WT BlALS). This was consistent with the higher concentration of acetoin synthesized by BlALS^N210D–H399D^, which means it had a lower sensibility to the acidic environment (Figure 4D). The results of enzymatic activity and fermentation indicated that BlALS^N210D–H399D^ accumulated more product acetoin than WT BlALS during the fermentation stage in an acidic environment and benefited from the better acid tolerance, which demonstrated that the improvement in acid tolerance enlarged the application potential of acetolactate synthase in industry.

As a synthetase with great economic value, cALS has been optimized for its stability. As reported so far, several key residues of cALS of *B. subtilis* were mutated, which confirmed that the mutant Q424S had significantly improved thermo-stability and catalytic efficiency that was almost unchanged; by contrast, the efficiency of enzymatic activity of Q487S improved, and thermo-stability decreased significantly (Sommer et al., 2015). However, there are few reports about mutations of cALS to optimize acid tolerance, so our study provides a baseline for mutations that code for acid tolerance in cALS.

**FIGURE 5:**
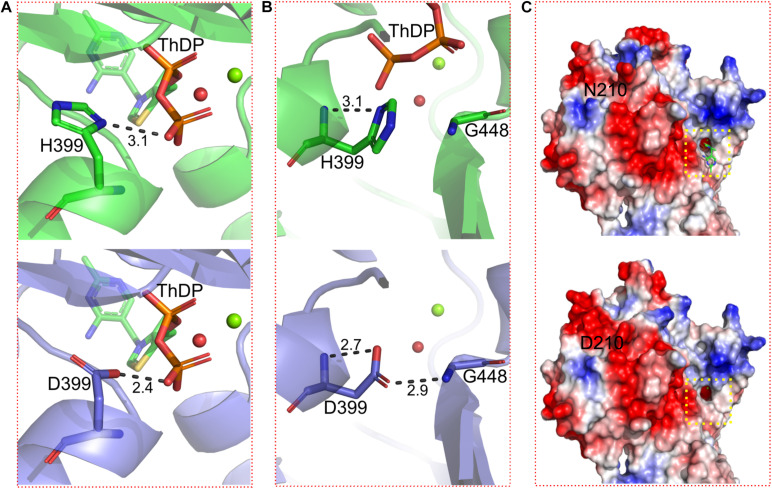
Details of conformational changes of WT BlALS and BlALS^N210D–H399D^. WT BlALS and BlALS^N210D–H399D^ are shown as cartoons colored in green and blue, respectively, and the related residues are shown as stick models. **(A)** Structural comparisons of the binding site for ThDP. **(B)** Structural comparisons of residue 399. **(C)** Comparisons of the surface electrostatic potential between WT BlALS and BlALS^N210D–H399D^, and the location of the active pocket is indicated by a red box.

To elucidate the mechanisms to improve acid tolerance of BlALS^N210D–H399D^, the structures of BlALS and BlALS^N210D–H399D^ were modeled using SWISS-MODEL workspace and by using the crystal structure of cALS from *B. subtilis* (PDB ID: 4RJJ) as a template. To explain the differences in acid tolerance between BlALS^N210D–H399D^ and WT BlALS, we analyzed the surface electrostatic potential and the stability of local conformation of them. The results of electrostatic potential showed that the mutation BlALS^N210D–H399D^ significantly changed the charge distribution around the residue 210 and increased the distribution of positive charges, which may be helpful for BlALS^N210D–H399D^ to better adapt to an acidic environment (Figure 5C). As expected, the charge distribution around the active center did not change, which meant that BlALS^N210D–H399D^ had no significant effect on substrate binding and enzymatic activity (Figure 5C). The method of improving the stability of protein by changing the distribution of surface charges has been applied successfully in the modification of other enzymes, such as rAgaZC-1 and LipK107 (Zhang et al., 2014; Su et al., 2019).

Because H399 was located in the interior of BlALS, mutation H399D had no significant effect on the distribution of surface charges. However, H399D formed a new hydrogen bond interaction with G448, which improved local conformational stability (Figure 5B). As confirmed by previous research, increasing the number of hydrogen bonds promoted the stability of enzymes (Joshi et al., 2000; Le Nours et al., 2003; Yang et al., 2013). In addition, H399D was located near the active site, so the improvement of local stability had a positive impact on the stability of enzymatic activity.

**FIGURE 6:**
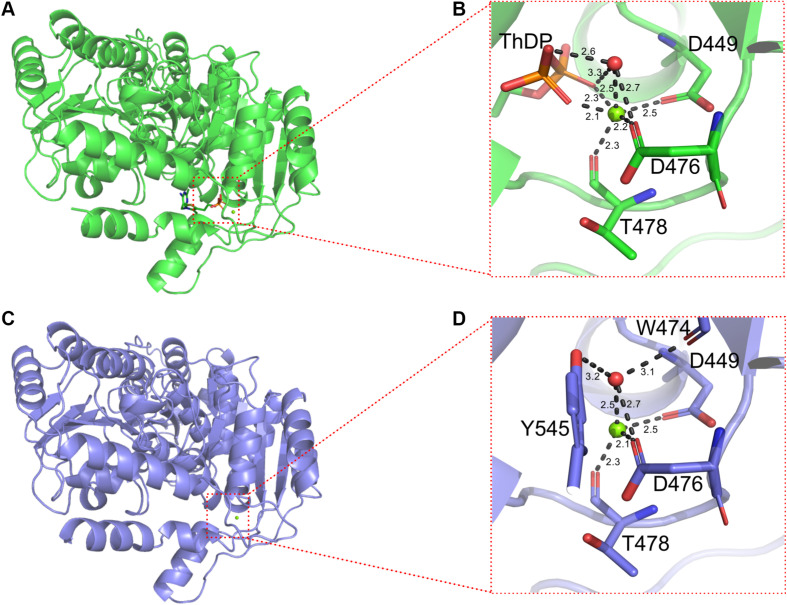
Details of binding sites of Mg^2+^ of WT BlALS and BlALS^N210D–H399D^. WT BlALS and BlALS^N210D–H399D^ are shown as cartoons colored in green and blue, respectively, and the related residues are shown as stick models. **(A)** Structural overview of WT BlALS. **(B)** Details of binding sites of Mg^2+^ of WT BlALS. **(C)** Structural overview of BlALS^N210D–H399D^. **(D)** Details of binding sites of Mg^2+^ of BlALS^N210D–H399D^.

In addition, ThDP was lost at the active site for BlALS^N210D–H399D^ (Figure 5C), so the coordination number of Mg^2+^ changed from 6 to 4 (Figure 6). The cofactors ThDP and Mg^2+^ were necessary for cALS to catalyze the decarboxylation of pyruvate and to transfer the generated hydroxyethyl combined with cofactors to another pyruvate to generate acetolactate (Pang et al., 2004). Due to the mutation of H399, the side chain of D399 clashed with the oxygen atom of the phosphoric acid group of ThDP, which made ThDP unable to be combined stably under the existing conformation (Figure 5A). However, due to the neutral condition formed by adding ThDP, BlALS^N210D–H399D^ still had enzymatic activity similar to WT BlALS (Figure 4A), which proved that it still had the ability to bind ThDP under a certain conformation; this is worth further study. In conclusion, with the acidic mutations N210D and H399D, the area of positive charge of the electrostatic potential on the protein surface and the number of hydrogen bonds near the active site increased, which was helpful for BlALS^N210D–H399D^ to withstand the acidic environment.

The comparison of fermentation results between BlALS and BlALS^N210D–H399D^ showed that BlALS^N210D–H399D^ had better ability to produce acetoin in 48–60 h in an acidic environment, which indicated that its greater tolerance of acid improved the yield of acetoin under acidic conditions. In view of the fermentation process of many fermented products under acidic conditions, the mutation that improved acidic stability of cALS was beneficial to the biosynthesis of aroma and healthy substances, such as TTMP and acetoin, which will improve the aroma and quality of these fermented products. Brewing microorganisms, such as *Bacillus*, produce some spontaneous mutations due to continuous growth and reproduction during long-term fermentation of Chinese liquor, which is one of the Chinese specialty fermentation products. After “acid-tolerant mutations” occur, individuals with acid-tolerant mutations emerge and survive in low-pH environments, such as pit bottom mud and yellow pulp water due to “natural selection and adaptation” (Wang et al., 2015). The results of this directed mutation confirmed that cALS in *Bacillus* in Daqu strong flavor liquor improved its acid tolerance and TTMP production through artificial, accelerated natural evolution. This could provide theoretical guidance for domestication of industrial strains.

## Conclusion

In this study, we improved the acid tolerance of BlALS from *B. licheniformis* T2 by site-directed mutagenesis based on the structural model. According to biochemical and structural analysis, the acidic mutations N210D and H399D increased the positive charge area of the electrostatic potential on the protein surface and the number of hydrogen bonds near the active site, which was helpful for BlALS^N210D–H399D^ to withstand an acidic environment. The results of enzymatic activity and fermentation tests showed that the acid tolerance of BlALS^N210D–H399D^ improved significantly, which improved the aroma and quality of liquor. This study further proved the feasibility of directed mutation of industrial enzymes based on protein structure, which provides direction for the study of spontaneous mutations of microorganisms and their metabolism in traditional fermented foods under human intervention.

## Data Availability Statement

All datasets presented in this study are included in the article/Supplementary Material.

## Author Contributions

TZ and JL: conceptualization. SY and YY: methodology. YL: software JL, YL, and TZ: validation. TZ: formal analysis, investigation, data curation, and visualization. JL and ZP: resources. TZ, JL, and SY: writing—original draft preparation. JL and YL: writing—review and editing. JL and RZ: supervision. JL: project administration and funding acquisition. All authors have read and agreed to the published version of the manuscript.

## Conflict of Interest

The authors declare that the research was conducted in the absence of any commercial or financial relationships that could be construed as a potential conflict of interest.

## Supplementary Material

Supplementary Figure 1 *K*_m_ and V_max_ by Non-linear Fitting for the BlALS and BlALS^N210D–H399D^.

Supplementary Figure 2 Growth curve of BlALS and BlALS^N210D–H399D^ in fermentation.

Supplementary Table 1 Characteristics, sources, or references for the strains and plasmids used.

Supplementary Table 2 Primers used for WT and site-directed mutagenesis.

## Abbreviations

cALS: catabolic acetolactate synthase
MTD: medium-temperature Daqu
ThDP: thiamine diphosphate
TTMP: tetramethylpyrazine
WT: wild-type.
