# Supplementary material for: Structure-Based Design of Acetolactate Synthase From Bacillus licheniformis Improved Protein Stability Under Acidic Conditions
Source: Front Microbiol. 2020 Oct 27;11:582909. doi: 10.3389/fmicb.2020.582909 (PMC7652814; doi:10.3389/fmicb.2020.582909)
Supplement: Supplementary Figure 1 — Km and Vmax by Non-linear Fitting for the BlALS and BlALSN210D–H399D. [file Data_Sheet_1.docx]

Structure-based design of acetolactate synthase from *Bacillus licheniformis* improved protein stability under acidic conditions

Ting Zhao, Yuan Li, Siqi Yuan, Zhifu Peng, Jun Liu* and Rongqing Zhou*

**List of the supplementary data**

**Table S1.** Characteristics, source or reference of the strains and plasmids using in this study

**Table S2.** Primes used for WT and site-directed mutagenesis

**Table S1.** Characteristics, source or reference of the strains and plasmids using in this study

| Strains/plasmids | Characteristics | Source or reference |
| --- | --- | --- |
| *Bacillus licheniformis T2* | The source of alsS | This work |
| *E.coli* DH5a | Host for gene cloning |  |
| *E. coli DE3* | Host for gene expressio |  |
| *E. coli DE3*/ pEGX-6p-1 | *E. coli DE3* harboring plasmid pEGX-6p-1 | This work |
| *E. coli DE3*/ pEGX-6p-1- alsS | *E. coli DE3* harboring plasmid pEGX-6p-1- alsS | This work |
| pMD19-T | Cloning vector, 2,692 bp, AmpR, lacZ | TaKaRa |
| pEGX-6p-1 | *E. coli DE3*expression vector, AmpR | This work |
| pEGX-6p-1-alsS | A derivative of pEGX-6p-1, AmpR, harboring the alsSgene | This work |

**Table S2.** Primes used for WT and site-directed mutagenesis

| **Primers** | **Primer sequences 5′–3′** |
| --- | --- |
| P_WT_F | GCGGATCCTTGAATAATGTAGCCGCTAAAAATG |
| P_WT_F | CGCTCGAGTCAAGATTGCTTAGAGGCTTCTTTATTA |
| P_H58D_F  P_H58D_R  P_R71D_F  P_R71D_R  P_K75D_F  P_K75D_R  P_K135D_F  P_K135D_R  P_N210D_F  P_N210D_R  P_N210E_F  P_N210E_R  P_H305D_F  P_H305D_R  P_H399D_F  P_H399D_R | GTTTGCCGTGACGAGCAGAATG  GATCAATTCAGGCCCCTTGTCTTT  GTCGGAGACTTGACTGGAAAGC  TGCCGCCGCCATAAATG  GGAGACCCCGGTGTTTGCC  AGTCAATCGTCCGACTGCCG  ATTACGGATTATAGCGCAGAAGTG  CGGCTGAAACAACGCCG  GCGGACCTTCCTGTCGTGCT  ATTGTGAATTTTGGCGATGGC  GCGGAACTTCCTGTCGTGC  ATTGTGAATTTTGGCGATGGC  GGCGAACGAAGCGTGATTGAT  TTTTCCATTCCAAAAGACCG  TCCGATGCGATTTGGATGTCTA  GCCGATGTCGCAAGTCACC |

**
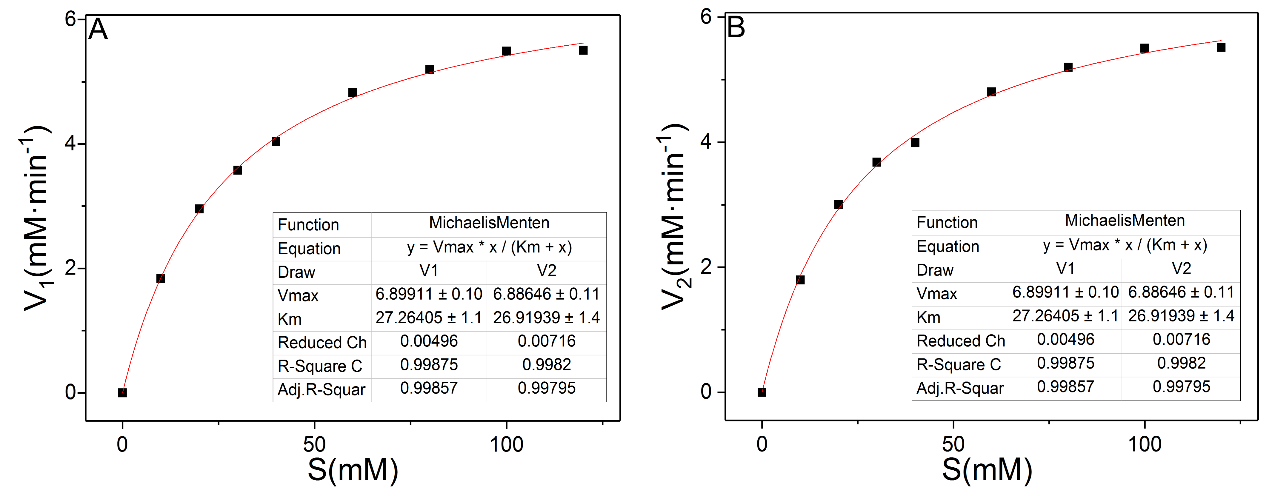
**

**Figure S1.** *K*_m_ and V_max_ by Nonlinear Fitting for the BlALS (A) and BlALS^N210D-H399D^ (B).


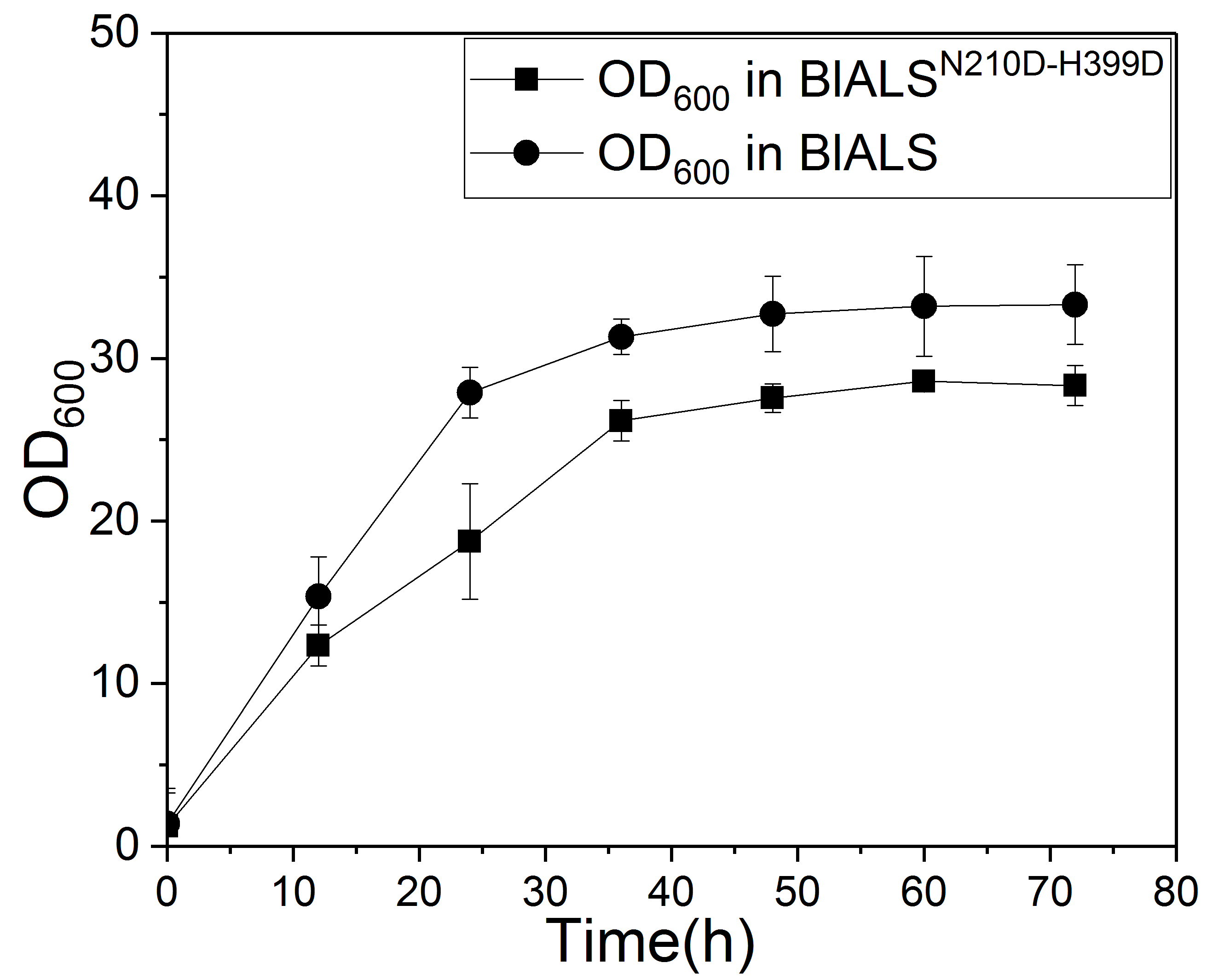


**Figure S2.** Growth curve of BlALS and BlALS^N210D-H399D^ in fermentation.
